# Precision immuno-oncology in oral cancer: latest trends in biomarkers, novel drug development and nanoparticle-based therapeutic platforms

**DOI:** 10.3389/fcell.2026.1839508

**Published:** 2026-06-03

**Authors:** Dominic Augustine, Samudrala Venkatesiah Sowmya, C. Pushpalatha, Kavitha Prasad, Hajira Khatoon

**Affiliations:** 1 Department of Oral and Maxillofacial Pathology and Oral Microbiology, Faculty of Dental Sciences, MS Ramaiah University of Applied Sciences, Bengaluru, Karnataka, India; 2 Department of Pediatric and Preventive Dentistry, Faculty of Dental Sciences, MS Ramaiah University of Applied Sciences, Bengaluru, Karnataka, India; 3 Department of Oral and Maxillofacial Surgery, Faculty of Dental Sciences, MS Ramaiah University of Applied Sciences, Bengaluru, Karnataka, India

**Keywords:** biomarkers, immunotherapy, nanomedicine, oral squamous cell carcinoma, precision immuno-oncology, programmed death-ligand 1, tumor immune microenvironment

## Abstract

Oral squamous cell carcinoma poses a significant global health burden, with over 370,000 annual cases and poor 5-year survival rates of 50%–60%, driven by risk factors like tobacco and alcohol. Despite advances in surgery, radiotherapy, and chemotherapy, functional morbidity and resistance necessitate precision immuno-oncology approaches. This review explores the tumour immune microenvironment in oral squamous cell carcinoma, characterized by immunosuppressive elements like M2 macrophages, myeloid-derived suppressor cells, and regulatory T cells, alongside spatial heterogeneity that complicates therapy. Biomarkers for patient selection include programmed death-ligand1 expression (via combined positive scoring), tumour mutational burden, neoantigen load, interferon-gamma-γ signatures, cytolytic scores, peripheral circulating tumour DNA, and single-cell/spatial profiling, though standardization remains critical. Immunotherapy has transformed oral squamous cell carcinoma management, with programmed cell death protein-1 inhibitors like nivolumab and pembrolizumab showing survival benefits in trials, particularly in programmed cell death protein-L1-positive cases. Emerging strategies encompass next-generation checkpoints (Lymphocyte activation gene-3, T-cell immunoreceptor with Ig and ITIM domains, OX40), personalized neoantigen vaccines, adoptive cell therapies (Tumour-Infiltrating Lymphocytes, Chimeric Antigen Receptor T-cell therapy), and rational combinations to counter resistance. Nanomedicine platforms—liposomes, polymeric nanoparticles, gold-based systems—enhance drug delivery, reprogram the Tumour and immune microenvironment, and enable chemo-immuno-photothermal synergies, addressing mucosal barriers and toxicity. Future priorities include biomarker validation via prospective registries, scalable Good Manufacturing Practice nanoplatforms, AI-driven multi-omic modeling, and federated learning for predictive analytics. By integrating tumour genomics, immune profiling, and advanced delivery, precision immuno-oncology holds promise to improve response rates, durability, and quality of life in oral squamous cell carcinoma.

## 1 Introduction

Oral squamous cell carcinoma (OSCC) constitutes more than 90% of malignancies arising in the oral cavity and represents a major global health challenge, particularly in low- and middle-income countries where exposure to tobacco smoking, alcohol, betel quid, and smokeless tobacco remains prevalent (Warnakulasuriya, 2009). According to recent global cancer statistics, oral cancer accounts for over 370,000 new cases and nearly 180,000 deaths annually (Sung et al., 2021; Johnson et al., 2020).

### 1.1 The clinical burden of OSCC: unmet needs and survival trends

Despite substantial advances in surgical techniques, radiotherapy delivery, and systemic treatment, improvements in long-term survival for oral squamous cell carcinoma have remained modest over recent decades with 5-year overall survival rates remaining approximately 50%–60% and outcomes remaining particularly poor in advanced and recurrent disease (Johnson et al., 2020; Ferris et al., 2016; Xue et al., 2025; De Graeff et al., 1999).

Moreover, molecular heterogeneity and adaptive resistance limit the durability of conventional cytotoxic therapies. These persistent unmet clinical needs underscore the urgency for biologically driven, less toxic, and more effective therapeutic strategies in OSCC (Leemans et al., 2018).

### 1.2 From broad immunotherapy to precision immuno-oncology: definitions and scope

The introduction of immunotherapy, particularly immune checkpoint inhibitors (ICIs) targeting the programmed death-1 (PD-1) and programmed death-ligand 1 (PD-L1) axis, has fundamentally altered the therapeutic landscape of head and neck squamous cell carcinomas, including OSCC. Landmark trials such as CheckMate-141 and KEYNOTE-048 demonstrated survival benefits with nivolumab and pembrolizumab in recurrent or metastatic disease, establishing immunotherapy as a new standard of care (Johnson et al., 2020; Ferris et al., 2019; Burtness et al., 2019; Bourhis et al., 2025).

Nevertheless, resistance to medications and immune related adverse effects linked to ICIs have restricted the clinical use in OSCC (Ru and Zheng, 2024). Translational studies of oral cavity tumours demonstrate marked heterogeneity in immune cell infiltration and functional immune states, which contribute to immune escape and therapeutic resistance (He et al., 2025; Mamilos et al., 2023). OSCC tumours employ multiple tumour-intrinsic and tumour-extrinsic mechanisms of immune suppression within the tumour microenvironment (Venkatesiah et al., 2022). This biological complexity limits the effectiveness of uniform therapeutic approaches and supports the need for precision-guided immuno-oncology strategies tailored to tumour- and host-specific immune characteristics.

Precision oncology refers to an approach to cancer treatment that is guided by the molecular, epigenetic, and phenotypic characteristics of an individual patient’s tumour, emphasizing the clinical relevance of tumour heterogeneity both within and between patients (Paliard and Rixe, 2019). Building on this concept, precision immuno-oncology has emerged as an integrative framework that tailors immunotherapeutic interventions according to tumour-intrinsic features and host immune context. This paradigm integrates tumour genomics, immune microenvironment profiling, spatial biology, and systemic biomarkers to refine patient selection, inform rational combination strategies, and improve the prediction of therapeutic efficacy and treatment-related toxicity (Xue et al., 2025; Wei et al., 2020).

Importantly, precision immuno-oncology also encompasses the development of enabling technologies—such as nanoparticle-based delivery systems and advanced computational models—to overcome biological barriers and enhance therapeutic efficacy. The incorporation of artificial intelligence, machine learning, and multi-omic data integration further expands the scope of this field by enabling predictive modeling of response, resistance, and immune-related adverse events (Wei et al., 2020; Li et al., 2024a).

PD-L1 is taken up into the cell through clathrin- and caveolin-dependent endocytosis and then directed to early endosomes, where it is either returned to the cell surface via Rab11-mediated recycling or marked for lysosomal degradation through ubiquitination. In oral squamous cell carcinoma, disruption of this balance favors persistent surface expression of PD-L1, enabling tumour cells to evade immune surveillance. In addition, PD-L1 can be packaged into exosomes formed within multivesicular bodies and released into the extracellular environment, thereby contributing to systemic immunosuppression and tumour–immune communication. Nanoparticles entering tumour cells are mainly internalized through endocytic pathways, including clathrin-mediated uptake, caveolae, or macropinocytosis, and subsequently traffic through endolysosomal compartments. Their ability to escape endosomes often depends on pH-responsive or surface-modified designs. Ultimately, the intracellular fate of these nanoparticles—whether recycled, degraded, or released into the cytosol—is governed by their size, charge, and surface characteristics. Notably, rational nanoparticle design can influence PD-L1 trafficking by promoting its degradation or limiting its exosomal release, thereby enhancing the effectiveness of immunotherapy in oral cancer (Gou et al., 2020; Ye et al., 2023).

Collectively, the transition from broad immunotherapy to precision immuno-oncology represents a fundamental shift toward individualized cancer care in OSCC. By aligning therapeutic strategies with tumour biology and immune contexture, this approach holds the potential to improve response rates, extend survival, and establish a more rational and sustainable framework for immunotherapy implementation in oral cancer.

## 2 Tumour and immune microenvironment (TIME) in oral cancer

Blood and lymph vessels, fibroblasts, endothelial cells, immune cells, cytokines, extracellular vesicles, and extracellular matrix are among the complex environmental elements that surround tumours. Tumour microenvironment (TME) is made up of all the stromal elements and the tumour cells (Wei et al., 2020). It is well known that the TME plays a major role in the onset, growth, and evolution of OSCC (Li et al., 2024a).

**FIGURE 1:**
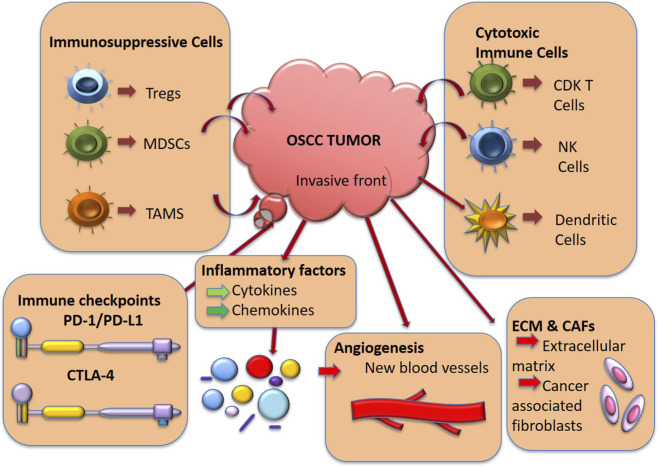
Tumor and immune microenvironment architecture in oral squamous cell carcinoma.

Tumour and immune microenvironment of OSCC plays a decisive role in disease progression, immune escape, and therapeutic response. Oral squamous cell carcinoma is characterized by a highly dynamic and heterogeneous microenvironment in which malignant cells interact with stromal and immune components to shape immunogenicity and resistance to therapy. Understanding the cellular composition, immune evasion mechanisms, and spatial organization of the TIME is central to the development of precision immuno-oncology strategies (Li et al., 2024a; Babu et al., 2024) (Figure 1).

### 2.1 Cellular components of the OSCC microenvironment

The oral squamous cell carcinoma TME is a complex ecosystem comprising malignant epithelial cells, cancer-associated fibroblasts, endothelial cells, and diverse immune populations including cytotoxic T lymphocytes, regulatory T cells (Table 1) (Venkatesiah et al., 2022).

**TABLE 1:** Key components of the immune microenvironment in OSCC.

Immune microenvironment component	Predominant phenotype/Characteristics	Functional role in OSCC progression
Tumour-Associated Macrophages	Predominantly M2-polarized macrophages	Promote tumour growth, angiogenesis, immune suppression, and extracellular matrix remodeling (Li et al., 2024a; Babu et al., 2024)
Myeloid-Derived Suppressor Cells	Immature myeloid cells with immunosuppressive activity	Secrete IL-10, and TGF-*β*, inhibit T-cell activation, and facilitate tumour progression (Babu et al., 2024)
T Lymphocytes	Reduced cytotoxic CD8^+^ T cells with enrichment of CD4^+^ regulatory T cells	Suppressed antitumour immune responses and impaired immune surveillance (Li et al., 2024a; Babu et al., 2024)
Cancer-Associated Fibroblasts	Activated fibroblasts within the tumour stroma	Remodel extracellular matrix and secrete pro-angiogenic factors such as VEGF and FGF, enhancing invasion and metastasis (Babu et al., 2024)
Tumour-Associated Neutrophils	N2-polarized neutrophils	Promote epithelial–mesenchymal transition, tumour invasion, and metastatic dissemination (Li et al., 2024a)

FGF, fibroblast growth factor; IL, interleukin; TGF-*β*, transforming growth factor beta; VEGF, vascular endothelial growth factor.

### 2.2 Mechanisms of immune evasion in OSCC

Oral squamous cell carcinoma employed multiple immune escape mechanisms, including PD-L1 upregulation, expansion of regulatory T cells, polarization of tumour-associated macrophages (TAMs) toward immunosuppressive phenotypes, and metabolic inhibition of effector T cells. PD-L1 expression in OSCC has been associated with aggressive clinicopathological features and variable responsiveness to immune checkpoint blockade. These immune escape processes are largely orchestrated by components of the tumour microenvironment, enabling tumour cells to evade effective immune surveillance. TAMs frequently acquire an M2-like phenotype that suppresses cytotoxic T-cell and natural killer cell activity through the secretion of anti-inflammatory cytokines such as interleukin-10 (IL-10) and TGF-*β*, thereby attenuating antitumour immunity. In parallel, myeloid-derived suppressor cells inhibit T-cell activation and proliferation through metabolic and oxidative mechanisms, including arginase activity and reactive oxygen species production. Additionally, tumour cells may evade immune recognition by downregulating major histocompatibility complex expression, impairing effective antigen presentation to cytotoxic T lymphocytes (Babu et al., 2024; Kannan et al., 2025).

### 2.3 Spatial heterogeneity and intratumoural evolution: implications for precision approaches

In addition to cellular composition, OSCC showed significant geographic heterogeneity and dynamic intratumoural evolution. These features have a significant impact on therapy response and restrict the precision of biomarker assessments based on a single sample. As a result of continuous clonal diversification and selection, multi-regional genomic and transcriptome investigations have shown significant variation in genetic changes, immune infiltration, and metabolic states across various tumour locations. Additional information about region-specific immune-suppressive niches and stromal interactions that contribute to treatment resistance is revealed by spatial transcriptome studies. The need for precision oncology techniques that incorporate multi-region sampling, geographical profiling, and longitudinal monitoring to more precisely capture tumour biology and direct tailored treatment plans is highlighted by these spatial and temporal complications (Mamilos et al., 2023; Steffen et al., 2024; Liu et al., 2024).

## 3 Biomarkers for patient selection and response prediction

The clinical efficacy of ICIs in OSCC is heterogeneous, with only a subset of patients achieving durable responses. Consequently, identifying reliable biomarkers capable of predicting therapeutic benefit and guiding patient selection has become a major priority in immuno-oncology research. Biomarkers reflecting tumour genomic characteristics, immune microenvironment composition, and systemic immune responses are increasingly being investigated to better understand determinants of immunotherapy sensitivity and resistance in OSCC and HNSCC cohorts (Ferris et al., 2016; Burtness et al., 2019).

Current biomarker strategies encompassed multiple biological layers. Tumour-based markers such as PD-L1 expression, assessed using immunohistochemical assays and standardized scoring systems, represent the most clinically validated predictors of response to PD-1/PD-L1 blockade in HNSCC (Saini et al., 2024). Genomic indicators including tumour mutational burden (TMB) and neoantigen load provide insight into tumour antigenicity and have been associated with improved responses to immune checkpoint inhibition across multiple cancer types, including head and neck cancers (Cristescu et al., 2018). Transcriptomic biomarkers, such as interferon-*γ*–related gene signatures and cytolytic activity scores, reflect immune activation within the TME and have shown predictive value for immunotherapy outcomes (Ayers et al., 2017).

In addition to tissue-based markers, peripheral blood biomarkers are increasingly being explored as minimally invasive tools for monitoring treatment response. These include circulating tumour DNA (ctDNA), immune cell phenotyping, and cytokine signatures, which may enable dynamic assessment of tumour evolution and immune responses during therapy (Wan et al., 2017). Advances in multiplex spatial profiling and single-cell sequencing technologies further allow high-resolution characterization of immune cell populations and their spatial organization within tumour tissues, providing deeper insights into tumour–immune interactions (Keren et al., 2018). Specifically, translation of these biomarkers into routine clinical practice requires careful attention to assay standardization, reproducibility, and regulatory validation, ensuring that biomarker-driven treatment decisions are robust and clinically meaningful (Simon et al., 2009).

Together, these complementary biomarker approaches provide a framework for precision immunotherapy in OSCC, with the potential to improve patient stratification, guide therapeutic selection, and inform the design of biomarker-driven clinical trials.

### 3.1 Programmed death-ligand 1 expression: assays, scoring systems, and predictive value in OSCC

Programmed death-ligand1 expression is currently the most clinically established biomarker for predicting response to PD-1/PD-L1 ICIs in OSCC and other HNSCC. It is typically evaluated using immunohistochemistry (IHC) assays performed on formalin-fixed paraffin-embedded tumour tissue. Several clinically validated antibody clones—including 22C3, 28–8, SP263, and SP142—are used across different diagnostic platforms, although variations in antibody specificity, staining platforms, and scoring methodologies can contribute to inter-assay variability (Noci et al., 2022). Efforts to harmonize PD-L1 testing across assays have therefore become an important aspect of biomarker standardization in immuno-oncology.

Two principal scoring systems are widely used to quantify PD-L1 expression in head and neck cancers: the tumour proportion score (TPS) and the combined positive score (CPS). The first one, TPS measures the percentage of PD-L1–positive tumour cells relative to all viable tumour cells, whereas CPS incorporates PD-L1 staining in both tumour cells and tumour-associated immune cells relative to the total number of tumour cells. Because immune cell PD-L1 expression contributes significantly to immune regulation in the TME, CPS is considered more reflective of tumour-immune interactions and has become the preferred scoring method in clinical trials evaluating immunotherapy in HNSCC (Bill et al., 2023).

The predictive value of PD-L1 expression for immunotherapy response in HNSCC has been demonstrated in several landmark clinical trials. In the pivotal KEYNOTE-048 phase III trial, pembrolizumab monotherapy significantly improved overall survival compared with cetuximab-based chemotherapy in patients with recurrent or metastatic HNSCC whose tumours exhibited PD-L1 CPS ≥20, establishing CPS-based PD-L1 assessment as a clinically actionable biomarker for treatment selection (Burtness et al., 2019; Rischin et al., 2021). Importantly, OSCC represented a substantial proportion of enrolled patients, supporting the applicability of PD-L1–based stratification in oral cancers. Similarly, the CheckMate-141 study evaluating nivolumab in platinum-refractory HNSCC demonstrated improved survival compared with standard chemotherapy, with exploratory analyses suggesting greater clinical benefit among patients with higher PD-L1 expression (Ferris et al., 2016).

Evidence from pooled analyses and cohort studies further supported the predictive role of PD-L1 expression. Meta-analyses evaluating PD-1/PD-L1 inhibitors across solid tumours, including HNSCC, have demonstrated that patients with elevated PD-L1 expression generally exhibit higher objective response rates and longer survival following immune checkpoint blockade (Levy et al., 2022). Nevertheless, clinical responses have also been observed in patients with low or negative PD-L1 expression, indicating that PD-L1 alone is an imperfect predictor of immunotherapy benefit.

In OSCC specifically, translational studies have reported heterogeneous PD-L1 expression across tumour specimens, reflecting the dynamic interplay between tumour cells and infiltrating immune populations. Some cohorts have demonstrated associations between elevated PD-L1 expression and increased tumour-infiltrating lymphocytes, suggesting adaptive immune resistance mechanisms within the TME (Lyford-Pike et al., 2013). Conversely, other studies have linked PD-L1 overexpression with aggressive clinicopathological features and poorer prognosis, highlighting the complex biological role of PD-L1 signaling in OSCC progression (Lin et al., 2015).

Overall, PD-L1 expression assessed using CPS currently represents the most clinically validated biomarker guiding immunotherapy use in OSCC and HNSCC. However, limitations related to spatial heterogeneity, temporal variability, and assay standardization underscore the need for integrating PD-L1 assessment with complementary biomarkers—such as TMB, immune gene expression signatures, and circulating biomarkers—to improve predictive accuracy in precision immuno-oncology.

### 3.2 Tumour mutational burden and neoantigen load: correlation with immunotherapy outcomes in oral cancer

Tumour mutational burden has emerged as an important genomic biomarker reflecting the total number of somatic mutations present within a tumour genome. High TMB increases the probability of generating neoantigens, which are tumour-specific peptides capable of being recognized by the host immune system. These neoantigens can enhance tumour immunogenicity by promoting T-cell recognition and activation, thereby improving the likelihood of response to immune checkpoint blockade therapies (Schumacher and Schreiber, 2015).

In head and neck cancers, including OSCC, genomic profiling studies have demonstrated a moderate to high mutational burden compared with many other solid tumours, largely driven by carcinogenic exposures such as tobacco and alcohol. Comprehensive genomic characterization performed by The Cancer Genome Atlas Research Network identified frequent somatic alterations and mutational processes in HNSCC, providing early evidence that these tumours harbor substantial neoantigen potential that could support immunotherapy responsiveness (Cancer Genome Atlas Network, 2015).

Clinical studies evaluating ICIs have further highlighted the relationship between TMB and therapeutic outcomes. In a pan-tumour analysis of patients treated with pembrolizumab, higher TMB levels were associated with significantly improved objective response rates and durable clinical benefit across multiple cancer types, including head and neck malignancies (Samstein et al., 2019). Subsequent analyses of patients enrolled in the KEYNOTE-012 and KEYNOTE-055 trials demonstrated that elevated TMB correlated with improved response to pembrolizumab in recurrent or metastatic HNSCC, supporting its role as a predictive biomarker for immune checkpoint blockade (Pfister et al., 2023).

In OSCC-focused translational studies, whole-exome sequencing has shown that tumours with higher mutational loads tend to exhibit increased infiltration of cytotoxic T lymphocytes and enhanced expression of immune-related genes, including INF-*γ*–associated signaling pathways. These findings suggested that TMB may serve as a surrogate marker for a pre-existing antitumour immune response in oral cancers (Patel et al., 2021; Zhang et al., 2026). Moreover, computational analyses integrating genomic and transcriptomic datasets have demonstrated that predicted neoantigen load correlates with immune activation signatures and T-cell infiltration in HNSCC cohorts containing substantial numbers of OSCC cases (Ren et al., 2019; Jiang et al., 2021).

Despite these promising observations, the predictive utility of TMB in OSCC remains less clearly defined than in other malignancies such as melanoma or lung cancer. Variability in sequencing platforms, differences in TMB calculation thresholds, and intratumoural genomic heterogeneity can complicate cross-study comparisons (Ning et al., 2022; Elbehi, 2024). Furthermore, some OSCC tumours with relatively low mutational burden may still respond to immunotherapy due to alternative mechanisms of immune activation, including viral antigens or inflammatory microenvironmental signals (Foy et al., 2020).

Consequently, current evidence suggested that TMB and neoantigen load represent complementary biomarkers rather than standalone predictors of immunotherapy response in OSCC. Integrative biomarker strategies that combine genomic metrics with PD-L1 expression, immune gene signatures, and spatial immune profiling are increasingly being explored to improve patient stratification and optimize the clinical use of immunotherapies in oral cancer.

### 3.3 Gene expression signatures and immune-related transcriptional profiles (IFN-*γ*), cytolytic scores

Beyond single biomarkers such as PD-L1 or TMB, immune-related gene expression signatures have emerged as powerful tools to characterize the functional state of the tumour immune microenvironment in OSCC. Transcriptomic profiling enables simultaneous assessment of multiple immune pathways—including interferon signaling, antigen presentation, cytotoxic activity, and T-cell activation—thereby providing a systems-level view of tumour–immune interactions. Such signatures are increasingly used to predict responsiveness to ICIs and to stratify patients in clinical trials.

One of the most extensively studied biomarkers is the IFN-*γ*–related gene expression signature, which reflects the presence of a T-cell–inflamed TME. In a seminal transcriptomic study by Mark Ayers and colleagues, an IFN-*γ*–related mRNA profile consisting of genes involved in antigen presentation, chemokine signaling, and cytotoxic T-cell activation was shown to predict response to PD-1 blockade across multiple tumour types, including HNSCC. The study demonstrated that tumours with high IFN-*γ* signature scores exhibited enhanced immune activation and were significantly more likely to respond to pembrolizumab therapy (Ayers et al., 2017). Subsequent analyses of patients enrolled in the KEYNOTE-012 cohort confirmed that IFN-*γ*–regulated genes—including *CXCL9, CXCL10, IDO1, HLA-DRA, STAT1,* and *IFNG*—were associated with improved progression-free survival following PD-1 blockade in HNSCC (Mehra et al., 2018).

Complementary evidence from transcriptomic analyses of large patient cohorts further supported the prognostic significance of immune gene signatures in head and neck cancers. In a study integrating data from The Cancer Genome Atlas HNSCC cohort, immune transcriptional profiles—including IFN-*γ*–associated genes, cytotoxic T-lymphocyte signatures, and antigen presentation pathways—were correlated with improved overall survival and favorable clinical outcomes. These signatures included genes such as *CD8A, CD8B, GZMB, PRF1,* and *CXCR6*, which collectively represent active cytotoxic T-cell responses within the TME (Johnson et al., 2020). Such findings suggest that immune-inflamed transcriptional phenotypes may identify OSCC tumours with pre-existing antitumour immunity that are more likely to benefit from immunotherapy (Mandal et al., 2016; Saiz-Ladera et al., 2021).

Another widely used transcriptomic metric is the cytolytic activity score, which quantifies the expression of effector molecules such as granzyme A (GZMA) and perforin (PRF1). This score reflects the cytotoxic capacity of infiltrating T lymphocytes and natural killer cells. Analyses of head and neck cancer datasets have demonstrated that tumours with high cytolytic scores display increased immune infiltration and enhanced expression of immune checkpoint molecules, including PD-1 and Lymphocyte activation gene-3 (LAG-3), indicating an active but potentially exhausted immune microenvironment (Rooney et al., 2015). Importantly, these immune-inflamed tumours are often more responsive to checkpoint blockade therapies.

Recent translational studies have further highlighted the clinical relevance of immune transcriptional profiling. For example, analyses of tumour samples from patients receiving combined immunotherapy regimens have demonstrated that cytokine and chemokine signatures linked to IFN-*γ* signaling—particularly *CXCL10* (IP-10)—are associated with improved clinical outcomes in head and neck cancers (Berszin et al., 2022). These findings reinforced the concept that immune gene signatures capture the dynamic interaction between tumour cells and the immune microenvironment, providing valuable predictive information beyond conventional histopathologic biomarkers.

Gene expression signatures representing IFN-*γ* signaling, cytotoxic T-cell activity, and immune activation pathways provide a robust framework for predicting immunotherapy responsiveness in OSCC. As high-throughput transcriptomic technologies become increasingly integrated into clinical research, these multi-gene biomarkers may play a central role in biomarker-driven trial design and personalized immunotherapy strategies for oral cancer.

### 3.4 Peripheral blood biomarkers: circulating tumour DNA, immune cell phenotyping, and cytokine signatures

Peripheral blood–based biomarkers are gaining increasing attention as minimally invasive tools for monitoring tumour burden, treatment response, and immune dynamics in OSCC. Unlike tissue-based assays that capture a single spatial and temporal snapshot of tumour biology, liquid biopsy approaches enable serial monitoring during treatment, which is particularly valuable for patients receiving immunotherapy. Peripheral biomarkers—including ctDNA, circulating immune cell subsets, and soluble cytokines—have therefore been investigated in prospective clinical cohorts of HNSCC, including oral cavity tumours (Wan et al., 2017; Ruiz-Torres et al., 2025).

Circulating Tumour DNA represents tumour-derived DNA fragments released into the bloodstream through apoptosis and necrosis of malignant cells. Advances in next-generation sequencing have enabled sensitive detection of tumour-specific mutations in plasma, providing opportunities for early detection of recurrence and monitoring of therapeutic response. In a prospective study of patients with HNSCC, plasma ctDNA profiling demonstrated high concordance with tumour genomic alterations and allowed detection of minimal residual disease after treatment (Wang et al., 2015). Another prospective analysis showed that ctDNA levels correlated with disease burden and could identify recurrence earlier than conventional imaging in patients with head and neck cancers (Silvoniemi et al., 2023). These findings suggest that ctDNA analysis may serve as a dynamic biomarker for treatment monitoring and early relapse detection in OSCC.

In addition to tumour-derived DNA, immune cell phenotyping in peripheral blood provides insight into systemic immune responses during immunotherapy. Flow cytometry-based studies in patients with recurrent or metastatic HNSCC treated with PD-1 inhibitors have shown that responders often exhibit higher baseline frequencies of activated CD8^+^ T cells and effector memory T-cell subsets. Conversely, elevated levels of immunosuppressive cell populations—such as regulatory T cells (Tregs) or myeloid-derived suppressor cells (MDSCs)—have been associated with poorer clinical outcomes (Asquino et al., 2025; Schumacher et al., 2024). Prospective immune-monitoring analyses have also demonstrated that early expansion of proliferating PD-1^+^ CD8^+^ T cells in peripheral blood may serve as a pharmacodynamic marker of response to PD-1 blockade (Sundström et al., 2024).

Circulating cytokines and chemokines represent another class of peripheral biomarkers reflecting systemic immune activation and inflammatory signaling. Prospective translational studies in patients with head and neck cancer receiving immunotherapy have identified interferon-γ–inducible chemokines—particularly CXCL9 and CXCL10—as potential predictors of treatment response. Elevated baseline levels of these cytokines are often associated with T-cell–inflamed TMEs and improved responsiveness to PD-1 blockade (Zhao et al., 2022). Conversely, increased circulating levels of pro-inflammatory cytokines such as IL-6 have been linked to treatment resistance and poorer survival outcomes in several patient cohorts (Jinn et al., 2015).

These studies demonstrate that peripheral blood biomarkers provide complementary information to tissue-based assays, capturing both tumour-derived genomic alterations and systemic immune dynamics. Integrating ctDNA analysis with immune cell phenotyping and cytokine profiling may enhance real-time monitoring of treatment response and facilitate early identification of resistance mechanisms in OSCC. As prospective biomarker-driven clinical trials continue to expand, liquid biopsy approaches are likely to play an increasingly important role in precision immunotherapy for oral cancer.

### 3.5 Multiplex spatial biomarkers and single-cell profiling: integrating tissue architecture with cell state

Recent advances in single-cell sequencing and spatially resolved molecular profiling have significantly improved the understanding of tumour–immune interactions in OSCC. Conventional bulk transcriptomic analyses average signals across heterogeneous tumour cell populations and immune infiltrates, potentially masking biologically relevant cellular subsets. In contrast, single-cell RNA sequencing (scRNA-seq) and multiplex spatial profiling technologies enable high-resolution characterization of individual cell populations while preserving information about their spatial organization within the TME (Manogaran et al., 2025; You and Liu, 2025). These approaches provide critical insights into how immune cells, stromal components, and malignant epithelial cells interact to influence disease progression and therapeutic responsiveness.

Single-cell transcriptomic studies of HNSCC have revealed extensive cellular heterogeneity within tumours, including diverse malignant cell states and complex immune cell populations. A landmark single-cell analysis conducted by Maximilian Puram and colleagues characterized thousands of cells from primary HNSCC tumours, including oral cavity cancers, and identified distinct malignant cell programs associated with partial epithelial-to-mesenchymal transition (p-EMT), immune modulation, and stromal interactions. Importantly, the p-EMT program was associated with increased invasiveness and adverse clinical outcomes, highlighting how transcriptional cell states can influence tumour behavior and patient prognosis (Puram et al., 2017).

Beyond tumour cell heterogeneity, single-cell profiling has also uncovered diverse immune cell populations within the OSCC microenvironment. Studies analyzing tumour-infiltrating lymphocytes have identified exhausted CD8^+^ T-cell subsets characterized by high expression of immune checkpoint molecules such as PD-1, LAG-3, and TIGIT (T-cell immunoreceptor with Ig and ITIM domains). These exhausted T-cell populations frequently coexist with immunosuppressive myeloid cells and regulatory T cells, forming a complex immune ecosystem that can limit effective antitumour immunity (Zheng et al., 2021). Such findings provide mechanistic insights into why only a subset of patients respond to immune checkpoint blockade and support the development of combination immunotherapy strategies targeting multiple immune regulatory pathways.

Spatially resolved profiling technologies further extend these insights by mapping immune cell populations within their anatomical context. Multiplex immunohistochemistry and spatial transcriptomics platforms enable simultaneous detection of multiple protein or RNA markers while preserving tissue architecture. Spatial analyses of HNSCC specimens have demonstrated that the proximity between cytotoxic T cells and tumour cells is a critical determinant of effective immune responses. Tumours with dense CD8^+^ T-cell infiltration within the tumour parenchyma—often referred to as “inflamed” or “hot” tumours—tend to exhibit better responses to immunotherapy compared with immune-excluded or immune-desert phenotypes (Ch et al., 2025; Li et al., 2024b).

Recent spatial transcriptomic studies have also highlighted the importance of TAMs and cancer-associated fibroblasts (CAFs) in shaping the immune landscape of OSCC. These stromal components can form physical and biochemical barriers that restrict T-cell infiltration and promote immunosuppressive signaling pathways. By integrating spatial localization with gene expression data, investigators have demonstrated that specific stromal niches enriched with immunosuppressive macrophages and fibroblasts correlate with poor immune infiltration and unfavorable clinical outcomes (You and Liu, 2025; Ch et al., 2025).

As these technologies, including single-cell and spatial profiling technologies, become more widely adopted in translational research, multiplex spatial biomarkers and single-cell profiling are expected to play an increasingly important role in biomarker discovery and precision immunotherapy for oral cancer.

### 3.6 Practical considerations: assay standardization, reproducibility, and regulatory pathways for clinical biomarker implementation

Despite rapid progress in biomarker discovery for OSCC, translating candidate biomarkers into routine clinical practice remains challenging. Successful clinical implementation requires robust assay standardization, reproducibility across laboratories, and clear regulatory validation pathways. Without these elements, even biologically promising biomarkers may fail to achieve clinical adoption (Zhao et al., 2026; Cheng et al., 2014).

One of the major challenges in biomarker implementation is analytical variability across diagnostic platforms. For example, assays used to measure PD-L1 expression—including antibody clones such as *22C3, 28–8, SP263,* and *SP142*—differ in staining protocols, scoring algorithms, and interpretation criteria. Such variability can lead to inconsistent patient classification and treatment decisions. Harmonization efforts involving multicenter analytical comparisons have therefore been undertaken to evaluate concordance between different PD-L1 assays used in immunotherapy trials for HNSCC (Scheel et al., 2016). These studies emphasized the importance of standardized protocols and validated companion diagnostics to ensure reproducibility across clinical laboratories.

Another critical consideration is inter-laboratory reproducibility of molecular assays used to assess genomic and transcriptomic biomarkers. Techniques such as next-generation sequencing (NGS), RNA sequencing, and multiplex immunohistochemistry require rigorous quality control procedures to ensure consistent results. Large-scale genomic initiatives, including those led by The Cancer Genome Atlas Research Network, have demonstrated the feasibility of standardized molecular profiling across multiple institutions through harmonized protocols for sample processing, sequencing, and data analysis (Ayers et al., 2017). These efforts provide an important framework for biomarker validation in translational cancer research.

Beyond analytical validation, clinical validation and regulatory approval are essential steps before biomarkers can guide therapeutic decisions. Regulatory agencies such as the U.S. Food and Drug Administration have established frameworks for the development and approval of companion diagnostics that identify patients most likely to benefit from targeted or immune-based therapies. For instance, PD-L1 testing using validated immunohistochemistry assays has been approved as a companion or complementary diagnostic for ICIs in several cancers, including HNSCC (Dobbin et al., 2016; Ou et al., 2021). These regulatory pathways require demonstration of analytical validity, clinical validity, and clinical utility through prospective clinical studies.

Another emerging challenge involves standardization of advanced biomarker platforms, such as single-cell sequencing, spatial transcriptomics, and multiplex proteomic assays. While these technologies provide unprecedented insight into tumour biology, their clinical implementation is limited by technical complexity, high cost, and variability in computational analysis pipelines. International consortia and regulatory science initiatives are therefore working to establish guidelines for data quality, reproducibility, and reporting standards in high-dimensional biomarker research (Mohr et al., 2024; Pl et al., 2025).

In summary, while numerous candidate biomarkers have been identified for predicting immunotherapy response in oral cancer, their clinical utility depends on rigorous assay standardization, reproducibility across laboratories, and regulatory validation. Continued collaboration among academic researchers, clinical laboratories, regulatory agencies, and industry partners will be necessary to translate biomarker discoveries into reliable tools for precision oncology in OSCC.

## 4 Harnessing novel drug development in OSCC

The treatment landscape for OSCC has significantly changed with the advent of immunotherapy. However, the small percentage of patients who have long-term benefits from immune checkpoint inhibition has prompted the creation of innovative immunotherapeutic drugs, individualised approaches, and sensible combination regimens. Precision immuno-oncology principles are tightly aligned with OSCC drug development, which is increasingly influenced by tumour biology, immunological context, and resistance mechanisms.

### 4.1 Immune checkpoint inhibitors in OSCC: landmark trials and real-world evidence

The treatment landscape for recurrent and metastatic OSCC has been completely changed by immune checkpoint inhibition. This has been made possible by important clinical trials that first demonstrated efficacy in the larger HNSCC population, as well as later OSCC-specific studies that investigated neoadjuvant and real-world settings. Pembrolizumab and nivolumab, two agents that target PD-1, have shown significant survival advantages and tolerable safety profiles, leading to regulatory approvals and integration into clinical practice for advanced illness (Ferris et al., 2016; Burtness et al., 2019).

In patients with platinum-sensitive recurrent/metastatic HNSCC, the KEYNOTE-048 trial was a pivotal phase III randomised study that established pembrolizumab as the first-line standard of care, either alone or in conjunction with platinum-based chemotherapy. It significantly improved overall survival (OS) when compared to the EXTREME regimen, especially in PD-L1-positive patients (CPS ≥1 and CPS ≥20). In patients with recurrent or metastatic disease, these data supported the widespread approval of pembrolizumab and pembrolizumab with chemotherapy. Subgroup studies have confirmed these findings, indicating a consistent benefit in oral cavity subsites (Burtness et al., 2019; Gillison et al., 2022).

CheckMate-141 demonstrated that nivolumab significantly improved OS compared with investigator’s choice in platinum-refractory recurrent/metastatic HNSCC, leading to its approval in this population and providing a basis for extrapolation to OSCC, despite the lack of phase III data specifically restricted to OSCC. Together, these studies show that PD-1 inhibition can change treatment paradigms in PD-1-sensitive OSCC subgroups and increase survival above previous chemotherapy outcomes (Ferris et al., 2016; Gillison et al., 2022).

#### 4.1.1 Neoadjuvant and early-phase OSCC-specific evidence

Neoadjuvant PD-1 inhibition has been investigated in early phase trials for resectable OSCC. The viability of checkpoint blockade before definitive resection was demonstrated by phase II research of neoadjuvant nivolumab in patients with stage II–IVA OSCC, which exhibited an objective response rate of approximately 33% with favourable safety and no surgical delays (Knochelmann et al., 2021). Furthermore, nivolumab plus ipilimumab in the neoadjuvant environment may cause both radiologic and pathologic responses, according to randomised phase II evidence, albeit more extensive research is required to determine its exact function (Schoenfeld et al., 2020). These outcomes strengthen the idea that neoadjuvant immunotherapy possibly enhance antitumour immunity and enhance long-term results in locally advanced OSCC.

#### 4.1.2 Real-world evidence

Real-world clinical outcome studies complement randomized trial data by capturing greater patient heterogeneity outside controlled eligibility criteria (Table 2). Retrospective analyses of patients with recurrent or metastatic HNSCC—including oral cavity cancers—treated with pembrolizumab or nivolumab in routine practice report objective response rates (∼20–25%) and median survival outcomes consistent with pivotal phase III results, while highlighting the influence of clinical factors such as PD-L1 expression and host characteristics on response (Kim et al., 2020; Huber et al., 2025). These empirical findings support the usefulness of ICIs and guide safety and efficacy expectations in larger OSCC populations.

**TABLE 2:** Key trials and real-world evidence of ICIs relevant to OSCC.

Study/Setting	Population	Therapy	Key outcomes	Evidence source
KEYNOTE-048	Recurrent/metastatic HNSCC (incl. oral cavity subsets)	Pembrolizumab ± chemotherapy vs. EXTREME	Improved OS; survival advantage in PD-L1 CPS ≥1/20	Phase III randomized trial (practice-changing) (Burtness et al., 2019)
CheckMate-141	Platinum-refractory HNSCC	Nivolumab vs. standard therapy	Improved OS vs. chemotherapy	Phase III trial supporting PD-1 use (Harrington et al., 2017)
Neoadjuvant nivolumab	Resectable OSCC	Nivolumab (neoadjuvant)	ORR ∼33%; safe and feasible	Phase II OSCC-specific study (Knochelmann et al., 2021)
Nivolumab ± ipilimumab (Phase 2)	Resectable oral cavity SCC	Nivolumab ± ipilimumab	Feasible; radiologic and pathologic responses	Phase II randomized study (Schoenfeld et al., 2020)
Real-world clinical cohorts	Recurrent/metastatic HNSCC (oral cavity included)	Pembrolizumab/Nivolumab	ORR ∼20–25%; OS/PFS consistent with trials	Retrospective real-world analyses (Kim et al., 2020; Huber et al., 2025)

CPS, combined positive score; HNSCC, head and neck squamous cell carcinoma; OSCC; oral squamous cell carcinoma; OS, overall survival; ORR, objective response rate; PDL-1, Programmed Death-Ligand 1; PFS, Progression-Free Survival; PD-1, Programmed Cell Death Protein 1.

Recent phase III trials, KEYNOTE-689 and NIVOPOSTOP, have added meaningful evidence in favor of incorporating immunotherapy into the curative treatment of locally advanced head and neck squamous cell carcinoma, including oral cavity cancers. In KEYNOTE-689, pembrolizumab was given around the time of surgery—both before (neoadjuvant) and after (adjuvant)—alongside standard surgery and tailored (chemo)radiotherapy. This approach led to a clear improvement in event-free survival and pathological response, along with a reduction in distant recurrences, suggesting that perioperative PD-1 blockade could become a new standard of care. In comparison, the NIVOPOSTOP trial focused on patients at higher risk of recurrence, using nivolumab after surgery in combination with postoperative chemoradiotherapy. This strategy resulted in better disease-free survival and improved loco-regional control compared to conventional treatment, highlighting the benefit of adding immunotherapy in selected high-risk cases (Burtness et al., 2022; Bourhis et al., 2025).

### 4.2 Next-generation checkpoints and co-stimulatory targets (LAG-3, TIGIT, OX40) — early-phase trial evidence

**FIGURE 2:**
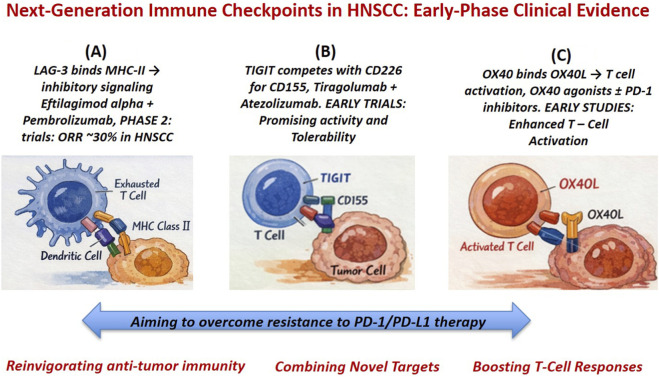
Next-Generation Immune Checkpoints in Head and Neck Squamous Cell Carcinoma: Early phase Clinical Evidence. **(A)** LAG-3. **(B)** TIGIT. **(C)** OX40.

To overcome resistance to PD-1/PD-L1 blockade, next-generation immune checkpoints and co-stimulatory pathways are being actively explored in early-phase clinical trials. Among these, inhibitory receptors such as LAG-3 and TIGIT, as well as the co-stimulatory receptor OX40 (CD134), have emerged as promising immunotherapeutic targets with translational relevance to HNSCC, including OSCC (Figure 2).

#### 4.2.1 Emerging inhibitory checkpoints beyond PD-1 and CTLA-4

Lymphocyte activation gene-3 is expressed on exhausted T cells and exerts inhibitory effects on antitumour T-cell responses by binding to Major Histocompatibility Complex Class II (Zaheer and Sureka, 2025). Soluble LAG-3 protein agonists that stimulate antigen-presenting cells, such as eftilagimod alpha (efti), have been evaluated in combination with the PD-1 inhibitor pembrolizumab in HNSCC. In the multicenter Phase II TACTI-002 study, efti plus pembrolizumab demonstrated an objective response rate (ORR) of approximately 30%, in patients with second-line recurrent/metastatic HNSCC and was well tolerated, with sustained increase in lymphocyte counts and Th1 cytokine biomarkers (Al-Batran et al., 2023; Forster et al., 2024; Immutep, 2024). Furthermore, in the TACTI-003 (KEYNOTE-PNC-34), Phase IIb trial evaluating first-line use in PD-L1–negative HNSCC, preliminary data showed a 26.9% ORR and a 57.7% disease control rate (DCR), supporting the potential of LAG-3 modulation to enhance immunotherapy responses in immunologically heterogeneous tumours (Al-Batran et al., 2023). Updated results from cohort analyses indicate even higher ORRs (≈35.5%) in certain patient subsets, with favorable safety profiles (Kristensen et al., 2024). These findings support the biological rationale for LAG-3 modulation as a strategy to reinvigorate anti-tumour immunity in immunotherapy-resistant disease.

#### 4.2.2 T-cell immunoreceptor with ig and ITIM domains

T-cell immunoreceptor with Ig and ITIM domains is an inhibitory immune checkpoint expressed on activated T cells and natural killer cells that suppresses I mmune effector function through competitive binding with the co-stimulatory receptor CD226 for shared ligands, including CD155 and CD112. Engagement of TIGIT skews immune signaling toward inhibition, contributing to T-cell exhaustion and impaired antitumour immunity within the TME (Ge et al., 2021). Given its role in immune exhaustion, TIGIT has emerged as an attractive target for combination immunotherapy strategies.

Early-phase clinical evaluation of TIGIT blockade has largely focused on the monoclonal antibody tiragolumab, administered alone or in combination with the PD-L1 inhibitor atezolizumab. In a Phase I/Ib dose-escalation study, tiragolumab demonstrated an acceptable safety and tolerability profile as monotherapy and in combination with atezolizumab in patients with advanced solid tumours, with no unexpected toxicities reported and observed favorable pharmacokinetic characteristics (Bendell et al., 2020). This study established the feasibility of dual TIGIT and PD-L1 blockade and provided the basis for further clinical development.

Subsequently, a Phase I/Ib study by Rodriguez-Abreu et al. evaluated tiragolumab alone and in combination with atezolizumab in patients with advanced solid tumours. The combination regimen was well tolerated, with manageable adverse events and evidence of sustained TIGIT receptor engagement and immune modulation. Importantly, preliminary signals of antitumour activity were observed with the combination compared with monotherapy, supporting the biological rationale for co-targeting TIGIT and PD-L1 pathways (Cho et al., 2022).

Further confirmation of safety and biological activity was provided by a Phase I study conducted in Japanese patients with advanced or metastatic solid tumours, in which tiragolumab plus atezolizumab demonstrated acceptable tolerability, predictable pharmacokinetics, and no dose-limiting toxicities. This study reinforced the consistency of the safety profile across different patient populations and supported global clinical development of TIGIT-based combinations (Yamamot et al., 2024).

Although these early-phase studies were not designed to assess definitive efficacy in OSCC, the favorable safety profiles, sustained target engagement, and preliminary antitumour signals observed across solid tumour cohorts provide strong justification for continued evaluation of TIGIT blockade. Overall, these findings support the incorporation of TIGIT inhibitors into rational combination immunotherapy strategies for head and neck cancers, particularly in the context of resistance to PD-1/PD-L1–based therapies.

#### 4.2.3 Co-stimulatory targets

Conversely, co-stimulatory agonists targeting receptors such as OX40 (CD134) aim to enhance T-cell activation and memory formation. OX40 (CD134) is a member of the tumour necrosis factor receptor (TNFR) superfamily that functions as a co-stimulatory receptor expressed predominantly on activated CD4^+^ and CD8^+^ T cells. Engagement of OX40 enhances T-cell proliferation, survival, cytokine production, and memory formation, thereby amplifying antitumour immune responses. Given its role as a positive immune regulator, OX40 has been explored as a therapeutic target using agonistic monoclonal antibodies in early-phase clinical trials (Goldman et al., 2022; Kim et al., 2022).

Early clinical evaluation of OX40 agonists has primarily focused on first-in-human Phase I and Phase Ib dose-escalation studies assessing safety, pharmacokinetics, and immunologic activity in patients with advanced solid tumours. In a Phase I study of the OX40 agonist antibody MEDI0562, treatment was generally well tolerated, with no dose-limiting toxicities observed across dose cohorts. Pharmacodynamic analyses demonstrated increased activation and proliferation of peripheral T cells, indicating effective engagement of the OX40 pathway (Goldman et al., 2022).

Similarly, MOXR0916, a humanized agonistic anti-OX40 antibody, was evaluated in a Phase I trial as monotherapy and in combination with the PD-L1 inhibitor atezolizumab in patients with advanced solid tumours. While clinical responses were limited, the study confirmed an acceptable safety profile and demonstrated immune activation, including increased T-cell infiltration and activation markers within the TME, supporting the biological activity of OX40 agonism in humans (Kim et al., 2022).

Additional early-phase studies have evaluated OX40 agonists such as BGB-A445 in combination with PD-1 blockade. These Phase I investigations demonstrated manageable toxicity profiles and pharmacodynamic evidence of T-cell activation, reinforcing the rationale for combination strategies aimed at coupling co-stimulatory signaling with immune checkpoint inhibition (Desai et al., 2025).

Although objective responses with OX40 agonists as monotherapy have generally been modest, the consistent safety profiles and evidence of immune engagement observed across early-phase trials suggest that OX40 agonism may be most effective when integrated into rational combination regimens. These findings provide a strong translational foundation for further exploration of OX40-based immunotherapy strategies in head and neck cancers, including OSCC, where enhancement of T-cell function remains a critical therapeutic goal.

### 4.3 Personalized cancer vaccines and neoantigen targeting: proof-of-concept trials and translational endpoints

Personalized cancer vaccines represent a transformative immunotherapeutic strategy designed to elicit tumour-specific immune responses by targeting patient-specific neoantigens arising from somatic mutations. Unlike shared tumour-associated antigens, neoantigens are absent from normal tissues, rendering them highly immunogenic and reducing the risk of central immune tolerance. Advances in NGS, bioinformatics, and vaccine platforms have enabled rapid identification and clinical targeting of neoantigens, leading to multiple proof-of-concept clinical trials across solid tumours (Zolkind et al., 2017; Xie et al., 2023).

Early landmark Phase I trials established the feasibility, safety, and immunogenicity of personalized neoantigen vaccines. A first-in-human study using a personalized neoantigen peptide vaccine (NeoVax) in patients with high-risk melanoma following surgical resection. The vaccine induced robust CD4^+^ and CD8^+^ T-cell responses against multiple neoantigens, with durable T-cell memory observed. Notably, vaccinated patients demonstrated delayed recurrence, and those who relapsed showed enhanced responses to subsequent PD-1 blockade, suggesting vaccine-induced immune priming (Ott et al., 2017; ClinicalTrials.gov, 2020).

Concurrently, Sahin et al. evaluated an individualized RNA-based neoantigen vaccine (RNA-LPX) in melanoma patients. This study demonstrated strong polyfunctional CD4^+^ and CD8^+^ T-cell responses directed against vaccine-encoded neoantigens, accompanied by trafficking of vaccine-specific T cells to tumour sites. Translational analyses confirmed epitope spreading and long-term immune persistence, validating neoantigen vaccines as potent inducers of antitumour immunity (Sahin et al., 2017).

More recent trials have focused on combining personalized cancer vaccines with ICIs to overcome tumour immune evasion. The Phase IIb randomized KEYNOTE-942 trial evaluated the mRNA-4157/V940 personalized neoantigen vaccine in combination with pembrolizumab versus pembrolizumab alone in patients with resected high-risk melanoma. The combination significantly improved recurrence-free survival and distant metastasis-free survival, providing the first randomized evidence that personalized neoantigen vaccines can confer clinical benefit when integrated with PD-1 blockade (Weber et al., 2024).

Translational endpoints across these studies have been central to demonstrating biological activity and mechanism of action. Key endpoints include neoantigen-specific T-cell frequency and functionality (measured by ELISPOT, intracellular cytokine staining, and TCR sequencing), clonal T-cell expansion, tumour infiltration of vaccine-reactive lymphocytes, and evidence of epitope spreading. These immune correlates have consistently shown concordance between vaccine-induced immune responses and clinical outcomes, reinforcing their value as surrogate biomarkers of efficacy (Ott et al., 2017; ClinicalTrials.gov, 2020; Sahin et al., 2017; Weber et al., 2024).

Although most clinical experience to date has been generated in melanoma and other solid tumours, the translational principles underpinning neoantigen vaccination are highly relevant to head and neck cancers, including OSCC, which exhibit moderate TMB and immunogenic potential. Ongoing efforts are now directed toward optimizing neoantigen prediction algorithms, accelerating manufacturing timelines, and integrating vaccines into multimodal immunotherapy regimens to maximize therapeutic benefit.

### 4.4 Adoptive cell therapies in OSCC

Adoptive cell therapies (ACT) represent a highly potent immunotherapeutic approach that directly augments antitumour effector immune populations through *ex vivo* expansion or genetic modification of autologous T cells. Key ACT modalities include tumour-infiltrating lymphocyte (TIL) therapy and genetically engineered T cells, such as chimeric antigen receptor (CAR) T cells (Chen et al., 2024). While ACT has demonstrated transformative efficacy in melanoma and hematologic malignancies, its clinical application in OSCC remains at an early developmental stage.

Tumour-infiltrating lymphocyte therapy involves the isolation and expansion of tumour-reactive lymphocytes from resected tumour tissue, followed by reinfusion after lymphodepleting chemotherapy. Durable clinical responses observed in melanoma established the foundational proof-of-concept for this strategy. Building on this success, early-phase studies have explored TIL therapy in head and HNSCC, including OSCC. In a landmark Phase II trial, Stevanović et al. demonstrated the feasibility of TIL therapy in heavily pretreated patients with metastatic HPV-associated epithelial cancers, including head and neck tumours, with objective responses and evidence of durable disease control in a subset of patients. These findings confirmed that functional tumour-reactive lymphocytes can be reliably isolated and expanded from HNSCC lesions, supporting further investigation of TIL-based approaches in OSCC (Stevanovic et al., 2019).

Despite encouraging feasibility, several challenges limit broader adoption of TIL therapy in OSCC, including variable lymphocyte yield, functional exhaustion of expanded T cells, and the profoundly immunosuppressive TME. Ongoing efforts are focused on optimizing TIL selection, enhancing persistence, and combining TIL therapy with ICIs to improve clinical durability (Stevanovic et al., 2019).

Chimeric antigen receptor T-cell therapy has achieved remarkable and durable clinical success in hematologic malignancies, particularly B-cell leukemias and lymphomas, where targeting lineage-restricted antigens such as CD19 has resulted in high response rates and long-term remissions (Maude et al., 2014; Neelapu et al., 2017); however, its application in OSCC remains investigational. To date, no completed clinical trials have evaluated CAR T-cell therapy exclusively in OSCC, and available evidence is derived from preclinical OSCC models and early-phase solid-tumour studies that included limited numbers of HNSCC patients. However, the translation of CAR T-cell therapy to solid tumours, including OSCC, has been significantly hindered by several biological barriers. These include marked antigen heterogeneity, inefficient trafficking and infiltration of CAR T cells into tumour sites, and a profoundly immunosuppressive TME characterized by inhibitory cytokines, metabolic constraints, and checkpoint ligand expression (Newick et al., 2017; Martinez and Moon, 2019).

Despite these challenges, multiple tumour-associated antigens relevant to HNSCC, including OSCC, have been explored as potential CAR targets. Epidermal growth factor (EGFR) is frequently overexpressed in HNSCC and has been extensively investigated due to its surface accessibility and oncogenic relevance (Sharafinski et al., 2010). Additional antigens such as HER2 and MUC1 have also been evaluated in preclinical models and early clinical studies, demonstrating antigen-specific recognition and cytotoxicity, albeit with concerns regarding on-target off-tumour toxicity.

### 4.5 Combination strategies to overcome primary and acquired resistance

Given the multifactorial mechanisms underlying resistance to ICIs, combination therapeutic strategies have become central to contemporary drug development in OSCC. Combining ICIs with chemotherapy or radiotherapy provides a strong biological rationale, as these modalities can induce immunogenic cell death, enhance tumour antigen release, upregulate major histocompatibility complex (MHC) expression, and promote dendritic cell–mediated immune priming. Clinical trials in HNSCC, which includes OSCC, have demonstrated that such combinations can improve response rates and yield clinically meaningful survival benefits in selected patient populations, particularly in the recurrent or metastatic setting (Ferris et al., 2016; Burtness et al., 2019; Formenti and Demaria, 2013).

Immune checkpoint inhibitor combinations with targeted agents, including anti-angiogenic therapies and EGFR inhibitors, aim to modulate the TME by normalizing aberrant tumour vasculature, reducing hypoxia, and alleviating immune suppression. Preclinical and translational studies suggest that vascular endothelial growth factor and EGFR signaling pathways contribute to T-cell exclusion and dysfunction, providing a mechanistic basis for combining these agents with immunotherapy (Allen et al., 2017; Concha-Benavente and Ferris, 2017). Early-phase clinical studies in HNSCC support the feasibility of these approaches, although optimal sequencing and patient selection remain areas of active investigation.

Beyond cytotoxic and targeted therapies, integrating ICIs with cancer vaccines or adoptive cell–based therapies seek to amplify tumour-specific immune responses and enhance the durability of clinical benefit. Therapeutic vaccines may expand antigen-specific T-cell repertoires, while adoptive cell therapies can overcome endogenous immune deficits, potentially synergizing with checkpoint blockade (Ott et al., 2017; Chen et al., 2024; Martinez and Moon, 2019). Although most randomized evidence has been generated in broader HNSCC populations, these immunobiological principles are directly applicable to OSCC, which shares key molecular drivers and immune-evasive features. Careful patient selection and biomarker-driven trial design will be essential to maximize therapeutic efficacy while minimizing toxicity in future OSCC-focused studies.

### 4.6 Safety, biomarkers of toxicity, and management of immune-related adverse events

Immune checkpoint inhibitors (ICIs) are now standard systemic therapies for recurrent and metastatic oral squamous cell carcinoma (OSCC) (Postow et al., 2018; Das et al., 2018). Given that many OSCC patients present with baseline mucosal inflammation, dysphagia, and poor nutrition, understanding immune-related adverse events (irAEs) is particularly important (Kha et al., 2020). Most safety data are derived from head and neck squamous cell carcinoma (HNSCC) trials with substantial oral cavity representation. In the CheckMate-141 trial, nivolumab showed a favorable safety profile, with grade ≥3 irAEs in about 13% of patients. Similarly, KEYNOTE-048 demonstrated acceptable safety of pembrolizumab, alone or with chemotherapy, with consistent toxicity profiles across tumor subsites. Common irAEs include dermatologic, endocrine, gastrointestinal, and hepatic effects, with severe events occurring in approximately 10%–15% of patients (Brahmer et al., 2018).

In OSCC, toxicities such as mucositis, dysphagia, and fatigue may cause greater functional impairment, especially when ICIs are combined with radiotherapy or chemotherapy, warranting close monitoring. Mechanistically, irAEs arise from immune dysregulation and loss of self-tolerance, with potential biomarkers including B-cell depletion, activated CD8^+^ T cells, elevated cytokines, and autoantibodies. Management follows ASCO and ESMO guidelines, emphasizing early detection and corticosteroid therapy for moderate to severe events. With appropriate management, ICIs remain safe and effective in OSCC, highlighting the need for improved, disease-specific toxicity prediction strategies (Haanen et al., 2017).

These data support that ICIs are clinically manageable and safe in OSCC when administered within structured toxicity-monitoring frameworks. Ongoing efforts to develop OSCC-specific toxicity biomarkers and integrate real-world safety data are essential to optimize patient selection and improve therapeutic outcomes.

## 5 Nanoparticle-based therapeutic platforms to overcome cancer resistance

Nanoparticle-based therapeutic platforms are increasingly being explored to address major therapeutic challenges in oral squamous cell carcinoma (OSCC), including inefficient drug delivery, systemic toxicity associated with conventional therapies, and the presence of a highly immunosuppressive tumour microenvironment (TME). By enabling targeted delivery, controlled drug release, and the co-transport of multiple therapeutic agents, nanomedicine approaches provide opportunities to enhance treatment efficacy while minimizing off-target effects. Consequently, nanoparticle systems are being investigated as versatile platforms to improve chemotherapy, immunotherapy, and combination treatment strategies in OSCC (Zhang et al., 2024). The following subsections summarize the rationale, types, immunomodulatory roles, and translational considerations of nanoparticle-based approaches in OSCC.

### 5.1 Rationale for nanomedicine in OSCC

Nanomedicine enables improved pharmacokinetics and targeted accumulation of therapeutic agents within tumour tissues through mechanisms such as the enhanced permeability and retention effect and ligand-mediated targeting of tumour-associated receptors (e.g., EGFR), which are frequently overexpressed in OSCC (Blanco et al., 2015). These properties allow higher intratumoural drug concentrations while minimizing off-target exposure.

In addition to enhancing drug delivery, nanoparticle systems can be engineered to modulate the OSCC TME, which is characterized by hypoxia, stromal barriers, and immunosuppressive cellular populations such as TAMs and myeloid-derived suppressor cells that limit effective antitumour immunity. Nanoparticles can deliver immunomodulatory agents, nucleic acids, or small molecules that reprogram these suppressive components, thereby promoting immune activation and improving responsiveness to immunotherapy (Jain et al., 2026).

Particularly, nanomedicine platforms facilitate the development of combination therapeutic strategies, which are increasingly recognized as necessary for effective OSCC treatment. Multifunctional nanocarriers can co-deliver chemotherapeutic agents, targeted inhibitors, and immunomodulators, enabling synchronized delivery and enhancing therapeutic synergy. Such systems may also incorporate stimulus-responsive release mechanisms that respond to tumour-specific conditions, further improving treatment precision and efficacy (Shi et al., 2017).

### 5.2 Types of nanoparticles

Several classes of nanoparticles have been explored as therapeutic and drug-delivery platforms in OSCC, including liposomes, polymeric nanoparticles, inorganic nanoparticles, and bioinspired metal systems (Table 3). Liposomal formulations improve chemotherapeutic delivery and reduce systemic toxicity, while polymeric nanoparticles such as Poly (lactic-co-glycolic acid) and chitosan systems enable sustained release and enhanced mucosal retention. Inorganic nanoparticles, particularly gold-based platforms, offer multifunctionality by combining drug delivery with photothermal and radiosensitizing properties. Emerging bioinspired nanoparticles, such as selenium-based systems, demonstrate intrinsic anticancer activity through oxidative stress–mediated apoptosis (Stathopoulos and Boulikas, 2012).

**TABLE 3:** Types of nanoparticles studied in OSCC.

Nanoparticle class	Representative example/Payload	Model (in vitro/*In vivo*)	Key findings in OSCC
Liposomes	Liposomal cisplatin (Lipoplatin-like formulations)	OSCC cell lines, xenograft models	Enhanced tumour accumulation, reduced nephrotoxicity, improved cytotoxic efficacy compared with free cisplatin (Shi et al., 2017; Stathopoulos and Boulikas, 2012)
	Curcumin-loaded liposomes	OSCC cell lines	Improved bioavailability, increased apoptosis, suppression of NF-κB signaling (Yallapu et al., 2012)
Polymeric nanoparticles	PLGA nanoparticles loaded with docetaxel	OSCC xenograft models	Sustained drug release, improved antitumour efficacy, reduced systemic toxicity (Danhier et al., 2012)
	Chitosan-based nanoparticles	OSCC cell lines	Enhanced cellular uptake, mucoadhesive properties suitable for oral cavity delivery (Danhier et al., 2012; Rizeq et al., 2019)
Inorganic nanoparticles– Gold (AuNPs)	Gold nanoparticles (bare or drug-conjugated)	OSCC cell lines, animal models	Induced apoptosis, enhanced radiosensitization and photothermal ablation (Dykman and Khlebtsov, 2012; Hainfeld et al., 2008)
	Cisplatin–gold–chitosan nanocomposites	OSCC cell lines	Synergistic cytotoxicity, improved intracellular cisplatin delivery (Ahmed et al., 2025)
Inorganic nanoparticles– Other	Mesoporous silica nanoparticles	OSCC cell lines	High drug-loading capacity, controlled release, enhanced anticancer efficacy (Mamaeva et al., 2013)
	Iron oxide nanoparticles	Preclinical OSCC models	Potential for image-guided therapy and hyperthermia-based tumour control (Estelrich et al., 2015)
Bioinspired/Metal nanoparticles	Selenium nanoparticles	OSCC cell lines	ROS-mediated apoptosis, anti-proliferative and anti-inflammatory effects (Karunakar et al., 2025)

NF-κB, Nuclear Factor kappa-B; OSCC, oral squamous cell carcinoma; PLGA, Poly(lactic-co-glycolic acid); ROS, reactive oxygen species.

### 5.3 Immunomodulatory nanoplatforms

Nanoparticle-based immunomodulatory platforms have emerged as promising strategies to enhance antitumour immunity in OSCC and other head and neck malignancies. These systems enabled targeted delivery of tumour antigens, immune adjuvants, and immunomodulatory agents, thereby improving antigen presentation, dendritic cell activation, and cytotoxic T-cell priming. In addition, nanoparticle platforms can modulate the TIME by reprogramming immunosuppressive cell populations such as TAMs and myeloid-derived suppressor cells, which are key mediators of immune evasion in OSCC. A growing body of *in vitro* and *in vivo* preclinical studies has demonstrated that nanoparticle-enabled antigen delivery, adjuvant co-delivery, and macrophage reprogramming strategies can enhance antitumour immune responses and improve therapeutic efficacy in experimental cancer models. Representative studies illustrating these immunomodulatory approaches are summarized in Table 4 (Chen et al., 2016; Rodell et al., 2018; Riley et al., 2019; Adkins et al., 2021; Kroemer et al., 2013).

**TABLE 4:** Representative *in vitro* and *in vivo* studies of nanoparticle-enabled immunomodulation relevant to OSCC.

Immunomodulatory strategy	Nanoparticle platform	Experimental model	Key immunological outcome
Antigen delivery (nanovaccine platforms)	Self-assembling peptide–TLR7/8 nanoparticle vaccines	*In vitro* dendritic cell activation; *in vivo* murine tumour models	Enhanced antigen presentation, dendritic cell maturation, and strong CD8^+^ T-cell responses against tumour antigens (Lynn et al., 2020)
Adjuvant co-delivery with tumour antigens	Polymeric nanoparticles delivering antigens with TLR agonists	*In vitro* dendritic cell stimulation; *in vivo* vaccination models	Improved T-cell priming and durable antitumour immune responses compared with soluble vaccines (Irvine and Dane, 2020)
Photothermal-immunotherapy nanoplatforms	Immune-adjuvant loaded nanoparticles combined with photothermal therapy	*In vivo* murine tumour models including head and neck cancer models	Increased tumour-infiltrating CD8^+^ T cells and enhanced response to immune checkpoint blockade (Chen et al., 2016)
Reprogramming TAMs	TLR7/8 agonist-loaded polymeric nanoparticles	*In vitro* macrophage polarization; *in vivo* tumour models	Induced TAM repolarization from M2 to pro-inflammatory M1 phenotype, promoting antitumour immunity (Rodell et al., 2018)
Targeting myeloid-derived suppressor cells	Immunomodulatory nanocarriers delivering cytokines or immune modulators	*In vitro* immune cell activation; *in vivo* tumour models	Reduced myeloid-mediated immunosuppression and improved T-cell-mediated antitumour responses (Riley et al., 2019)

TLR, Toll-Like Receptor; TAMs, tumour-associated macrophages.

### 5.4 Nano-enabled combination approaches: delivering ICIs, chemo-immunotherapy payloads, and photothermal/photodynamic adjuncts

Building on the delivery advantages discussed in earlier sections, nanoparticle platforms are increasingly being investigated to facilitate combination therapeutic strategies in OSCC. These systems enable the integration of chemotherapy, immunotherapy, and photo-based modalities within a single therapeutic framework, thereby improving treatment precision and potentially enhancing antitumour immune responses.

**FIGURE 3:**
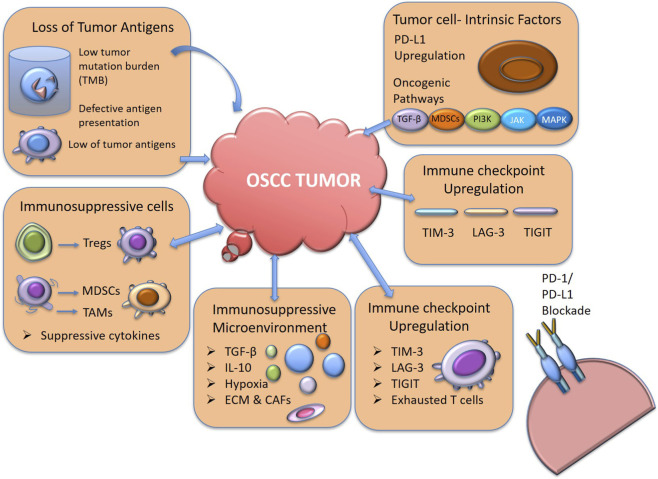
Mechanism of resistance to immune checkpoint inhibitors.

One clinically relevant example of nano-enabled chemotherapy is albumin-bound paclitaxel (nab-paclitaxel), a nanoparticle formulation designed to improve solubility and tumour delivery of paclitaxel. In a Phase II clinical study involving patients with recurrent or metastatic HNSCC, nab-paclitaxel combined with carboplatin demonstrated encouraging response rates and manageable toxicity, supporting the feasibility of nanoparticle-based chemotherapy in head and neck cancers (Rodell et al., 2018). Beyond direct cytotoxicity, taxane-based therapies can promote immunogenic cell death, increasing tumour antigen release and dendritic cell activation, thereby providing a biological rationale for combining such agents with ICIs (Figure 3) (Kroemer et al., 2013).

**FIGURE 4:**
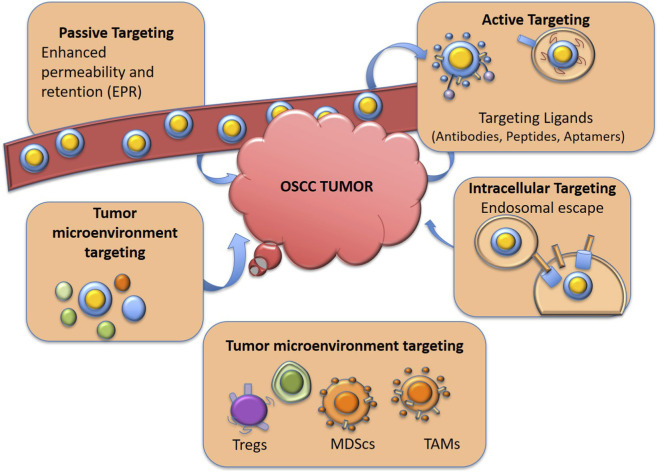
Nanoparticle delivery pathways.

Nanoparticle systems have also been explored for immunotherapy delivery and modulation of the TIME. Lipid nanoparticles and polymeric nanocarriers can deliver nucleic acids, immune adjuvants, or checkpoint-targeting molecules directly to tumour tissues, enhancing antigen presentation and T-cell activation. Advances in lipid nanoparticle technology—widely used for mRNA-based therapeutics—have demonstrated efficient delivery of nucleic acid payloads in humans, highlighting the translational potential of nanocarriers for immune-modulating therapies in solid tumours (Hou et al., 2021) (Figure 4).

In addition to drug delivery, nanoparticles enable photothermal therapy (PTT) and photodynamic therapy (PDT) as adjunct strategies capable of stimulating antitumour immunity. Clinical use of photodynamic therapy in head and neck cancers has demonstrated effective local tumour control and acceptable safety profiles, particularly in patients with recurrent or superficial lesions (Hopper, 2000). Mechanistically, photo-induced tumour destruction can trigger immunogenic cell death, releasing tumour antigens and promoting dendritic cell maturation, which may enhance systemic antitumour immune responses when combined with immunotherapy (Chen et al., 2016).

Finally, nano-enabled combination approaches offer a versatile strategy to integrate chemotherapy, immunotherapy, and phototherapy in OSCC treatment. Continued translational research and carefully designed clinical trials will be essential to determine optimal nanoparticle formulations and combination regimens capable of overcoming the immunosuppressive tumour microenvironment while maintaining favorable safety profiles.

### 5.5 Translational challenges: biodistribution, mucosal barriers, toxicity, and manufacturing considerations

Despite promising preclinical advances, the clinical translation of nanoparticle-based therapeutics in OSCC faces several biological and regulatory challenges. A key limitation is achieving effective biodistribution and retention within oral tumours. The oral cavity possesses unique anatomical and physiological features—including continuous saliva flow, rapid epithelial turnover, and complex vascular and lymphatic networks—that can limit nanoparticle residence time and reduce local drug accumulation (Patra et al., 2018). These factors can compromise the therapeutic efficiency of nanocarriers and necessitate the development of formulations capable of enhanced mucosal adhesion and sustained release.

Another important barrier is the oral mucosal interface, which acts as a protective biological barrier against foreign particles. Tight epithelial junctions, mucus layers, and enzymatic activity can restrict nanoparticle penetration and limit drug delivery to deeper tumour tissues (Ensign et al., 2012). Strategies such as mucoadhesive nanoparticles, surface-modified nanocarriers, and stimuli-responsive systems are being investigated to overcome these limitations and improve drug penetration into oral tumours.

Safety and toxicity considerations also remain central to clinical translation. While many nanoparticle formulations demonstrate favorable tolerability in preclinical models, systemic toxicity, immunogenicity, and long-term accumulation in organs such as the liver and spleen remain potential concerns in humans (Wilhelm et al., 2016). Clinical experience with approved nanomedicines, including liposomal doxorubicin and albumin-bound paclitaxel, has demonstrated improved pharmacokinetic profiles but also highlighted the importance of careful dose optimization and toxicity monitoring in patients (Barenholz, 2012).

In addition, manufacturing scalability and regulatory requirements represent significant hurdles for nanoparticle therapeutics. Reproducible large-scale production, quality control, and batch consistency are essential for regulatory approval. Regulatory agencies require detailed characterization of nanoparticle size, surface properties, stability, and pharmacokinetics to ensure safety and efficacy in clinical applications (Etheridge et al., 2013). These considerations are particularly important when developing multifunctional nanoplatforms that combine diagnostic and therapeutic capabilities.

Overall, overcoming these translational barriers will require multidisciplinary collaboration integrating materials science, oncology, and regulatory science. Continued clinical investigation and optimization of nanoparticle design are essential to realize the full potential of nanomedicine-based immunotherapy strategies in OSCC.

### 5.6 Limitations and challenges of PD-L1 as a marker for tumour mutational burden

PD-L1, despite its widespread use, is an unreliable standalone biomarker due to marked intra- and intertumoural heterogeneity and its dynamic regulation by cytokines such as IFN-γ. Differences in immunohistochemical assays, scoring methods (TPS vs. CPS), and cut-off values further compromise consistency and comparability across studies. Clinically, responses to immune checkpoint inhibitors are seen even in PD-L1–negative tumours, while many PD-L1–positive cases fail to benefit, underscoring its limited predictive value. Tumour mutational burden (TMB), proposed as an alternative, also shows inconsistent performance, as high TMB does not always correlate with response and is influenced by factors such as neoantigen quality and tumour immune context. In head and neck squamous cell carcinoma, overall response rates to immunotherapy remain modest, and major phase III trials like KEYNOTE-412 and JAVELIN Head and Neck 100 have not demonstrated significant survival benefits in unselected patients. A substantial proportion of patients still do not respond to PD-1/PD-L1 blockade, reflecting the complexity of the tumour microenvironment, including immune suppression and antigen escape. Similarly, adoptive cellular therapies such as CAR-T cells face significant hurdles in solid tumours, including antigen heterogeneity, poor tumour infiltration, and an immunosuppressive microenvironment. Together, these challenges highlight the need for integrated biomarkers and combination therapeutic approaches to improve outcomes in HNSCC (Ascione et al., 2025; Aboaid et al., 2025; Pfister et al., 2023).

## 6 Future directions and research priorities

Future progress in the management of oral squamous cell carcinoma (OSCC) will depend on translating emerging scientific insights into clinically applicable precision oncology strategies. Key priorities include systematic biomarker validation, scalable nanomedicine development, and integrative data-driven frameworks that can improve patient selection and therapeutic outcomes.

### 6.1 Prioritized biomarker validation studies and prospective registries

Advancing precision immunotherapy for OSCC requires systematic validation of predictive biomarkers in large prospective cohorts. Current biomarkers such as PD-L1 expression, TMB, and immune gene-expression signatures demonstrate variable predictive performance across HNSCC, including OSCC, highlighting the need for robust biomarker validation strategies (Burtness et al., 2019; Cristescu et al., 2018). Prospective clinical trials and registry-based observational studies can provide real-world data on treatment response, toxicity, and survival outcomes while enabling standardized biomarker assessment across institutions. Large collaborative initiatives, including genomic profiling programs such as The Cancer Genome Atlas, have already generated extensive molecular datasets for HNSCC and provide a valuable framework for integrating genomic, transcriptomic, and clinical data to guide biomarker discovery (Cancer Genome Atlas Network, 2015). Establishing dedicated OSCC registries that incorporate longitudinal clinical outcomes and biospecimen repositories will be essential for validating biomarkers that can guide patient selection for immunotherapy and emerging nanomedicine-based treatments.

### 6.2 Scalable nanomedicine platforms and GMP manufacturing pipelines

For nanoparticle-based therapeutics to transition from experimental studies to routine clinical practice, scalable and reproducible Good Manufacturing Practice (GMP) production platforms are required. Manufacturing consistency, particle stability, and batch-to-batch reproducibility represent critical determinants for regulatory approval of nanomedicine products (Etheridge et al., 2013). Lessons from clinically approved nanoparticle formulations—such as liposomal chemotherapeutics and albumin-bound drug formulations—demonstrate that standardized production processes can successfully support large-scale clinical use. Recent advances in lipid nanoparticle technology, which enabled the rapid clinical deployment of mRNA vaccines, highlight the feasibility of scalable nanomedicine manufacturing pipelines for therapeutic applications in oncology (Hou et al., 2021). Future research efforts should therefore focus on developing modular nanoparticle platforms that allow rapid incorporation of therapeutic payloads—including ICIs, nucleic acids, and antigenic peptides—while maintaining regulatory compliance and manufacturing scalability.

### 6.3 Data sharing, federated learning, and multi-omic integration for predictive modelling

The complexity of OSCC biology and immunotherapy response necessitates integrative data-driven approaches capable of analyzing large multi-dimensional datasets. Integration of genomic, transcriptomic, proteomic, and immunological data can enable the development of predictive models for treatment response and toxicity. Large-scale collaborative initiatives such as the Cancer Genome Atlas and the International Cancer Genome Consortium have demonstrated the value of multi-omic datasets in characterizing tumour heterogeneity and identifying molecular subtypes of head and neck cancers (Cancer Genome Atlas Network, 2015). Emerging computational frameworks—including federated learning approaches that allow secure data sharing across institutions without direct data transfer—offer promising solutions for collaborative analysis of sensitive clinical datasets (Rieke et al., 2020). These approaches can facilitate the development of predictive algorithms integrating imaging, molecular, and clinical variables, thereby enabling personalized treatment strategies for patients with OSCC.

## 7 Conclusion

The evolution from broad immunotherapy to precision immuno-oncology in OSCC addresses longstanding unmet needs amid modest survival gains and high morbidity from conventional therapies. Heterogeneous TIME dynamics, immune evasion mechanisms, and suboptimal biomarker performance underscore the imperative for tailored strategies leveraging PD-L1, TMB, transcriptomic signatures, and spatial technologies to optimize patient selection and predict responses.

Emerging paradigms—next-generation checkpoints, neoantigen vaccines, adoptive therapies, ICI combinations, and immunomodulatory nanoplatforms—offer synergistic potential to overcome primary/acquired resistance while minimizing toxicity. Translational challenges like mucosal barriers, manufacturing scalability, and irAE management demand multidisciplinary efforts, including prospective registries and AI-integrated multi-omics. Realizing precision immuno-oncology’s promise requires rigorous validation, fostering a paradigm shift toward biologically informed, individualized care that enhances survival and preserves function in OSCC patients.
